# Development of a Capacitive Ice Sensor to Measure Ice Growth in Real Time

**DOI:** 10.3390/s150306688

**Published:** 2015-03-19

**Authors:** Xiang Zhi, Hyo Chang Cho, Bo Wang, Cheol Hee Ahn, Hyeong Soon Moon, Jeung Sang Go

**Affiliations:** 1 School of Mechanical Engineering, Pusan National University, 2, Busandaehak-ro 63beon-gil, Geumjeong-gu, Busan 609-735, Korea; E-Mails: zxzx61@gmail.com (X.Z.); favre@naver.com (H.C.C.); wangbomems@gmail.com (W.B.); ach@pusan.ac.kr (C.H.A.); 2 Korea Institute of Industrial Technology, 60beon-gil, Gangseo-gu, Busan 618-230, Korea; E-mail: hsmoon@kitech.re.kr

**Keywords:** ice sensor, capacitance, temperature, ice growth

## Abstract

This paper presents the development of the capacitive sensor to measure the growth of ice on a fuel pipe surface in real time. The ice sensor consists of pairs of electrodes to detect the change in capacitance and a thermocouple temperature sensor to examine the ice formation situation. In addition, an environmental chamber was specially designed to control the humidity and temperature to simulate the ice formation conditions. From the humidity, a water film is formed on the ice sensor, which results in an increase in capacitance. Ice nucleation occurs, followed by the rapid formation of frost ice that decreases the capacitance suddenly. The capacitance is saturated. The developed ice sensor explains the ice growth providing information about the icing temperature in real time.

## 1. Introduction

The monitoring of ice growth is an important issue in aviation and transportation systems because ice can cause severe accidents. For instance an emergency landing of a British Airways Boeing 777 occurred in Gatwick airport on 17 January 2008. It was revealed that thick layers of ice originated from dissolved water in the fuel had built up within the fuel pipes as the aircraft passed over Siberia. As the aircraft approached the airport, this ice was released as a so-called “snow ball” which blocked the fuel flow at the fuel-cooled oil-cooler leading to a sudden loss of power. The problem occurred in the engines when the aircraft was close to the airport and it was unable to reach the runway. It crash-landed just short of the runway—thankfully without any casualties [1].

In a sub-zero temperature environment, the presence or formation of ice must be detected in real time. This will allow the pilot or driver to take appropriate action. However, there are no available ice detectors for directly detecting the thickness of ice within fuel pipes rather than just the presence of ice. This paper presents a cost-effective and small size capacitive ice sensor. It is also challenging to measure ice growth in real time. A specially designed environmental test system to simulate the sub-zero ice conditions is also presented.

## 2. Working Mechanism of the Ice Sensor

**Figure 1:**
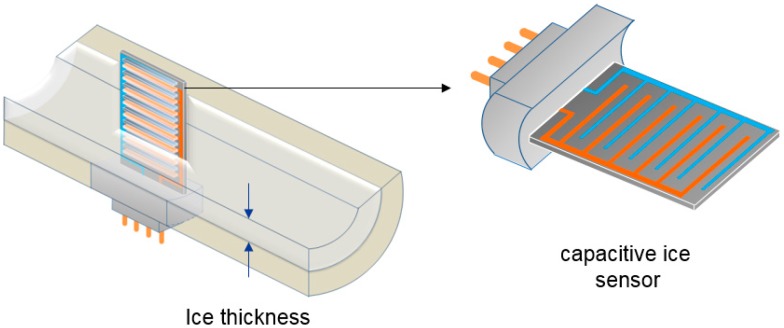
Schematic view of the capacitive ice sensor installed in a pipe to measure the ice growth from the inner surface of the fuel pipe.

Figure 1 shows a schematic drawing of a fuel pipe with the proposed capacitive ice sensor. The capacitive ice sensor can be installed by machining a hole in the fuel pipe. The ice starts to grow from the inner surface of the fuel pipe since the surface is colder than the fuel based on the heat conduction theory.

The capacitive ice sensor uses interdigitated comb electrodes, which have been widely used for electrostatic actuators [2], accelerometers [3] and frequency tuning filters [4]. The electrodes are paired to measure the ice growth. The capacitance can be obtained from Equation (1):

(1) $$C=\varepsilon_{o}\varepsilon_{r}\frac{A}{d}N$$

where $\varepsilon_{o}$ denotes the vacuum permittivity and $\varepsilon_{r}$, the relative static permittivity of a material, *d*, the distance between two confronting electrodes and *N*, the number of electrode pairs [5]. The dielectric constant of the vacuum is about 8.854 × 10^−12^ F/m, and the relative static permittivity for water is 80 and for the kerosene jet fuel it is 2.0 at 273 K and 4.2 for ice at 263 K in an input frequency of 10^5^ Hz [6].

As the ice grows, jet fuel in the medium surrounding the confronting electrodes is replaced with ice. In Equation (1), the relative static permittivity of the jet fuel is changed to that of the ice. As a result, the ice sensor detects in real time the change in capacitance, which is proportional to the increase in ice thickness.

## 3. Fabrication of the Ice Sensor

The ice sensor consists of paired electrodes on the front surface to measure the ice growth by using the capacitance change and a K-type thermocouple temperature sensor on the back surface to measure the temperature at which the ice forms. To examine the effect of the substrate on the ice growth, Pyrex 7740 glass with a low thermal conductivity of 1.05 W/mK and a silicon wafer with a high thermal conductivity of 149 W/mK were compared.

**Figure 2:**
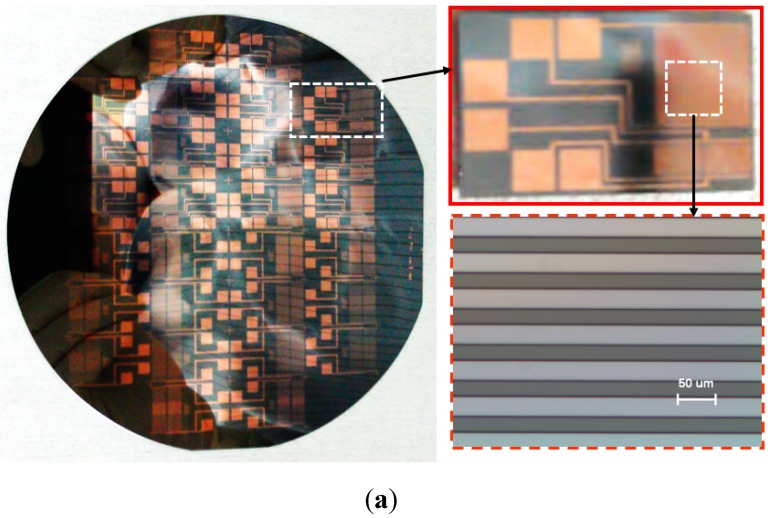

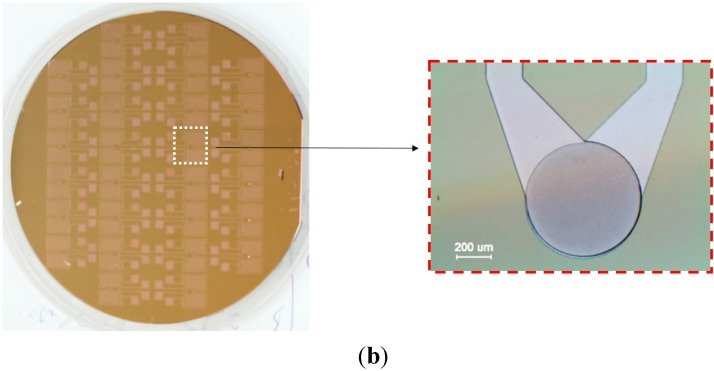
Fabricated ice sensor array on the silicon wafer. (**a**) Capacitive ice sensor (front); (**b**) Thermocouple (back)

Three masks were designed for fabrication. Firstly, the interdigitated comb-type electrodes for the capacitive sensor were made directly on the front surface by a standard image reversal process as shown in Figure 2a. The ice sensor was designed to measure the ice thickness from 0 to 6 mm. The gap distance of the pairing electrodes was 30 μm and their confronting width was 6 mm. Gold with a thickness of 3000 Å was deposited for the electrodes. Sixty (60) pairs of the electrodes were designed.

Secondly, to fabricate the K-type thermocouple temperature sensor, Alumel was patterned, followed by patterning of Chromel [7]. Both materials were sputtered on the surface of the patterned photoresistor, opposite to the surface containing the capacitive ice sensor. Figure 2b shows the fabricated thermocouple temperature sensor. The thermocouple junction was positioned at the center of the capacitive ice sensor. Its junction size was 600 μm. For the electrical interconnection, the terminal electrode pad was sized at 2.5 mm × 2.5 mm.

A feasibility test of the fabricated ice sensor was performed in water. The ice sensor was placed in a reservoir with a scale. As deionized water was poured into the reservoir and the level of water raised linearly from 0 to 6 mm with an increment of 1 mm, the capacitance change was measured for an input frequency of 100 kHz and a voltage of 1000 mV at an ambient temperature of 22 °C.

**Figure 3:**
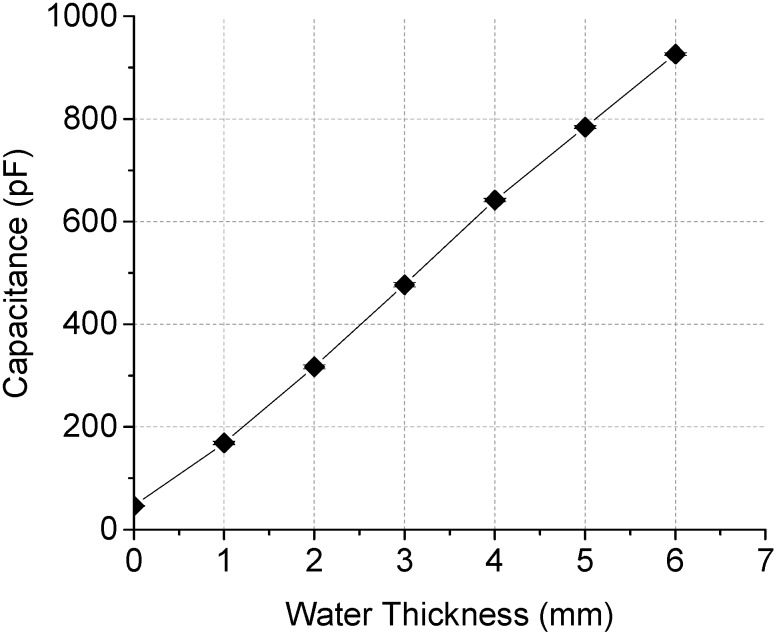
Measurement of capacitance for an increasing water height by using the fabricated ice sensor.

Figure 3 shows that the capacitance increases proportionally for the water level. This shows the linearity of the fabricated ice sensor. The comb-type electrodes are interdigitated along the height so that the output is also linear with respect to height.

**Figure 4:**
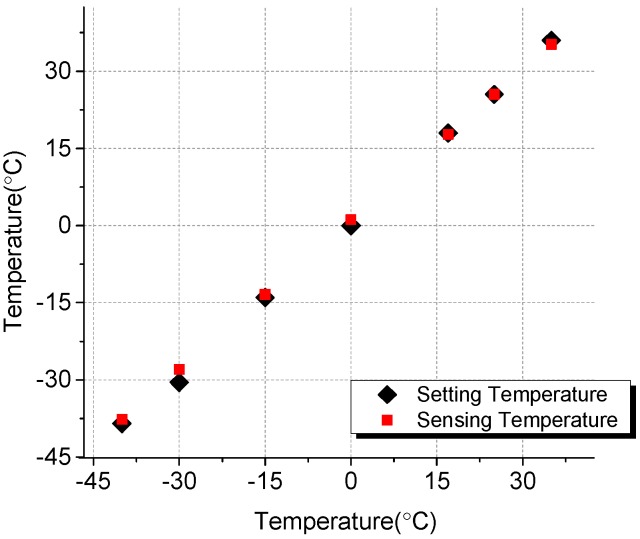
Temperature measured by the fabricated thermocouple.

Also, the fabricated K-type thermocouple temperature sensor was calibrated by comparing with a reference K-type thermocouple temperature sensor. In a thermal cycler, the environmental temperature was set from −40 °C to 50 °C, analogous to the temperature that an airplane might experience. As shown in Figure 4, the temperatures measured by the thermocouple in the fabricated ice sensor and the reference thermocouple were identical with less than 1% measurement error.

## 4. Environmental Test System for the Ice Growth

In the real engineering situation, the water is dissolved in the jet fuel and the ice nucleates on the inner surface of the fuel pipe. However, to examine the ice growth in the fuel pipe, the test environment requires an expensive and complicated setup for the accurate control of the amount of water in the fuel and temperature. Thus, the measurement was performed in air for the experimental evaluation.

**Figure 5:**
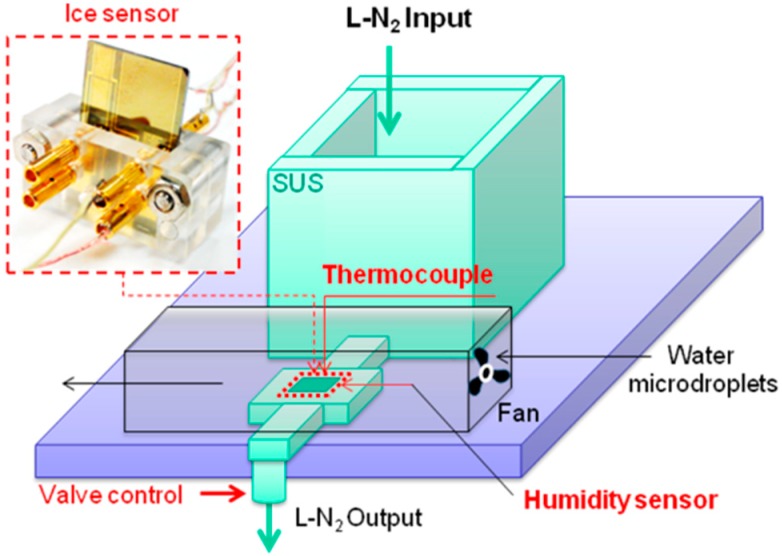
Cooling chamber to control temperature and humidity.

To nucleate the ice, humidity was used as a water source. Firstly, a commercial environmental chamber was tested to control the temperature and humidity. However, as soon as the ice started to form, the humidity changed in the chamber. Thus, for the feasibility test of the ice growth measurement in real time we specially designed an environmental test system, which can control temperature and humidity separately and simultaneously. Figure 5 shows a drawing of the environmental test system. It is composed of a reservoir of liquid nitrogen, a cold plate to form the ice, a humidifier with a fan. A duct guides water droplets generated from the humidifier to control the humidity.

In the experiment, the humidity was set to a targeted humidity as a water source to form ice on the sensor and then liquid nitrogen was flown to the cold plate to simulate an ice formation temperature of below 0 degrees. As the ice formed, the capacitance and the temperature of the ice formation were measured simultaneously.

**Figure 6:**
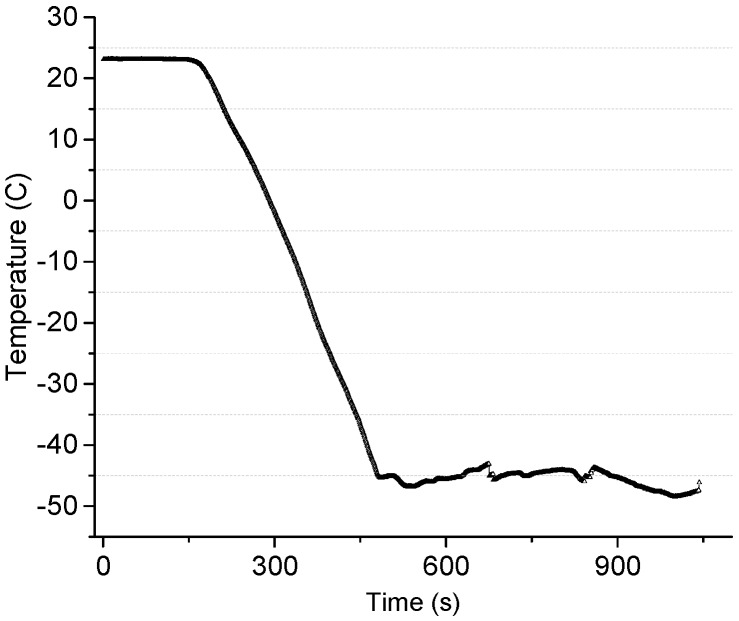
Cooling performance of the fabricated cooling chamber.

To control the temperature of the cold plate from ambient to −40 °C for the ice growth, liquid nitrogen (hereafter, LN2) with a boiling temperature of −195.79 °C at atmospheric pressure was used. The LN2 was filled in the reservoir with a volume of 1000 mL. The reservoir was connected with a sudden expansion-sudden contraction tube and the flow rate could be controlled by regulating the valve. The center of the extended part in the tube was machined to insert the ice sensor. The valve opening allowed maintaining the temperature at −45 °C for 30 min when the reservoir was fully charged. Figure 6 shows that the temperature could be maintained at −45 °C for 10 min after it dropped from the ambient temperature of 23 °C.

**Figure 7:**
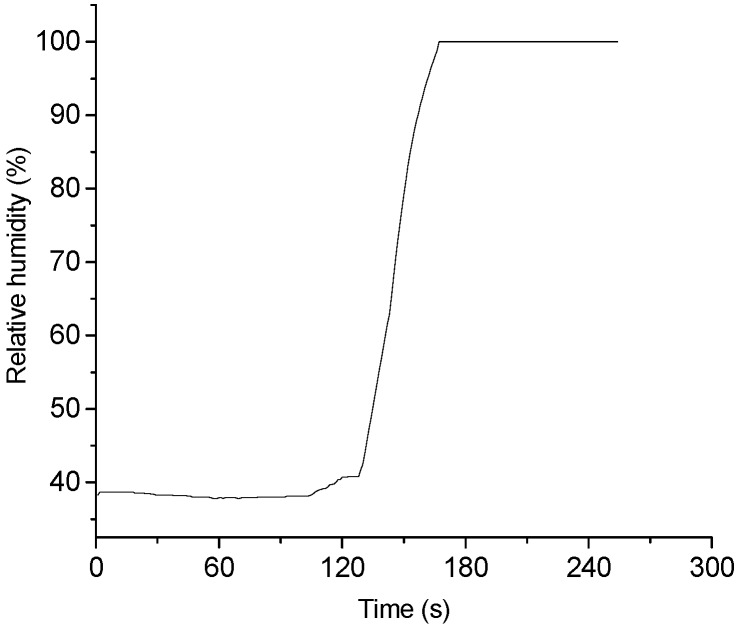
Environmental control performance of humidity in the cooling chamber.

The humidity must be constant while the ice grows. It is very difficult to use ambient humidity as an ice nucleation source, since it always changes. In order to obtain a constant relative humidity, a humidifier and a fan were used to introduce microdroplets into the test section. Here, the humidity was controlled by regulating the amount of microdroplets for the constant fan speed. Figure 7 shows that the relative humidity at 40% and 100% respectively is well controlled in the test section.

## 5. Measurement of Ice Growth

**Figure 8:**
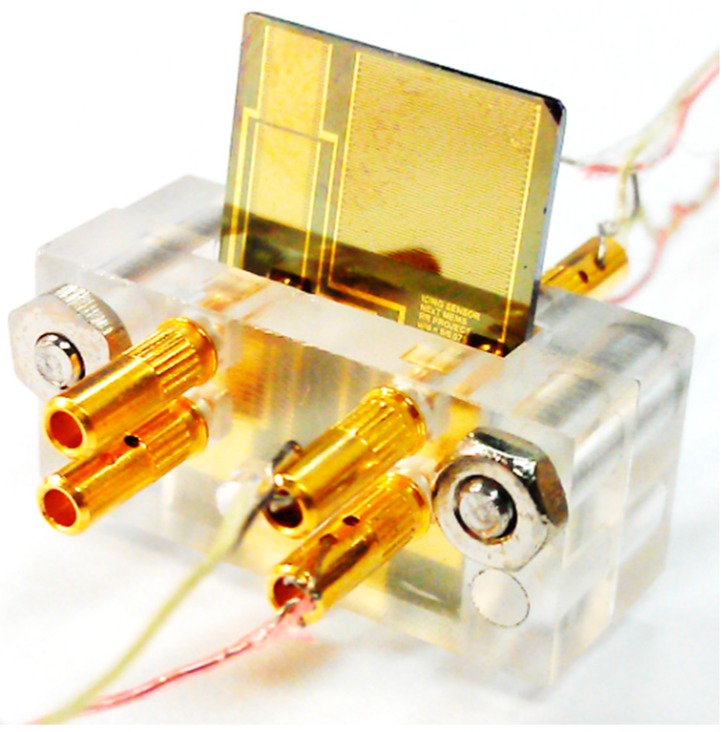
Picture of the plastic packaged ice sensor

Finally, real time measurement of the ice growth was performed. The capacitive ice sensor was packaged with external electrodes, as shown in Figure 8. The ice sensors fabricated on two different substrates were inserted into the cooling plate. The ice growth was examined as the ice grew from the surface of the cooling plate with the introduction of the water droplets. For the ice sensor on the silicon substrate, the entire surface of the ice sensor was covered with frost ice rather than dense ice due to its high thermal conductivity, whereas, the ice grew much faster from the cooling plate compared with ice from the ice sensor on the Pyrex glass owing to its low thermal conductivity. In this experiment, the ice sensor on the glass substrate was used to examine the measurement of the ice growth in real time.

The capacitance was measured for an AC input frequency using an LCR-meter. In the capacitance measurement, the relative permittivity depends on the temperature and the input frequency [8]:

(2) $$C=\frac{1}{2\pi fX_{c}}$$

where *f* is the input frequency and *X_c_* is the capacitance reactance.

In order to eliminate their effect on the capacitance measurement, various input frequencies were tested. To prevent ice formation on the capacitive ice sensor at subzero temperatures, it was wrapped with a plastic bag and charged with nitrogen gas to remove humidity. As reported in previous work [9,10], at the input frequency of more than 100 kHz, their effect was potentially negligible. The capacitance measured at an input frequency of 100 kHz was constant, even at the decreased temperature.

Finally, the ice growth was measured using a capacitive ice sensor on the glass substrate. After the ice sensor was installed in the cold plate, the LN2 valve was opened. When the temperature approached −45 °C, the humidifier and fan were turned on. The capacitance and temperature were measured from the sensor simultaneously over time.

**Figure 9:**
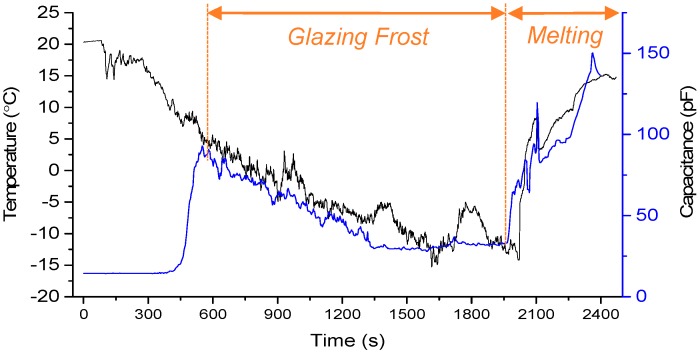
Real time measurement of ice formation cycle.

As shown in Figure 9, as the temperature decreased over time, the capacitance increased rapidly and at a threshold it decreased suddenly. Then the capacitance was saturated. Also, by melting the ice with an air blower, the capacitance was detected. The measured capacitance directly explains the situation occurring on the ice sensor.

The first increase in the capacitance could be explained by the formation of a water film on the ice sensor. The water droplets generated by the humidifier attached on the sensor surface and accumulated to form a liquid film by hydrophilicity. The air on the ice sensor was replaced by the liquid, resulting in an increase in the capacitance because the relative static permittivity of water is 80 times higher than that of air. More water droplets attached and the water thickness grew with time. As a result, the capacitance rose.

The sudden decrease in the capacitance could be explained by the formation of ice. The nucleation of ice started to occur and the frost ice grew quickly. Thus, the capacitance in the graph dropped sharply. Then, there was a general descent over time and temperature. The fluctuating decrease reflected the combination of the formation of frost and the attachment of water droplets.

**Figure 10:**
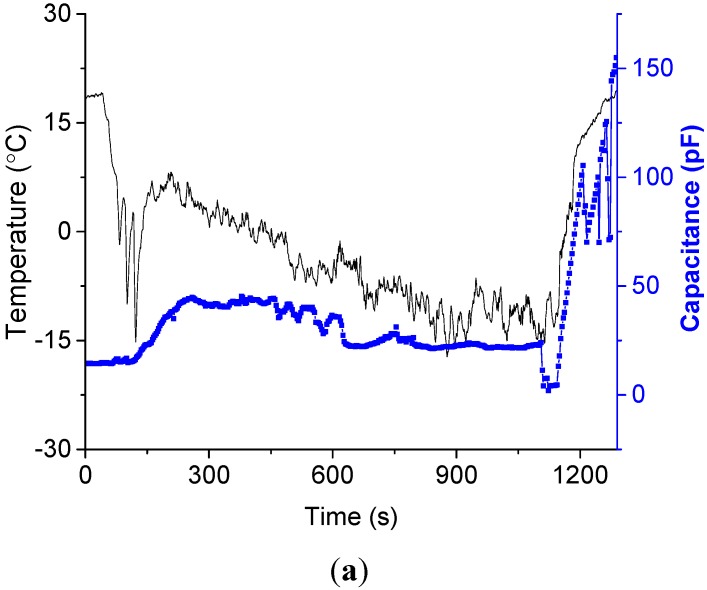

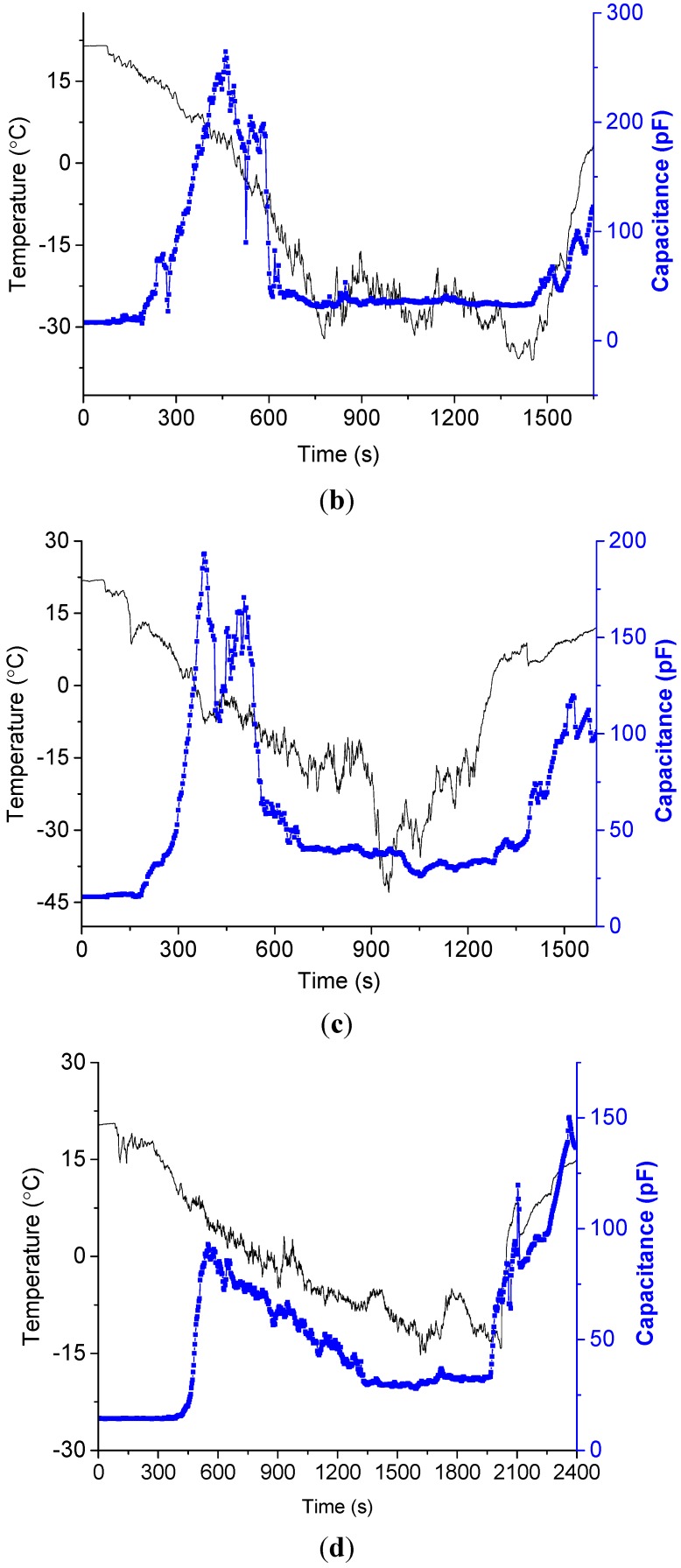

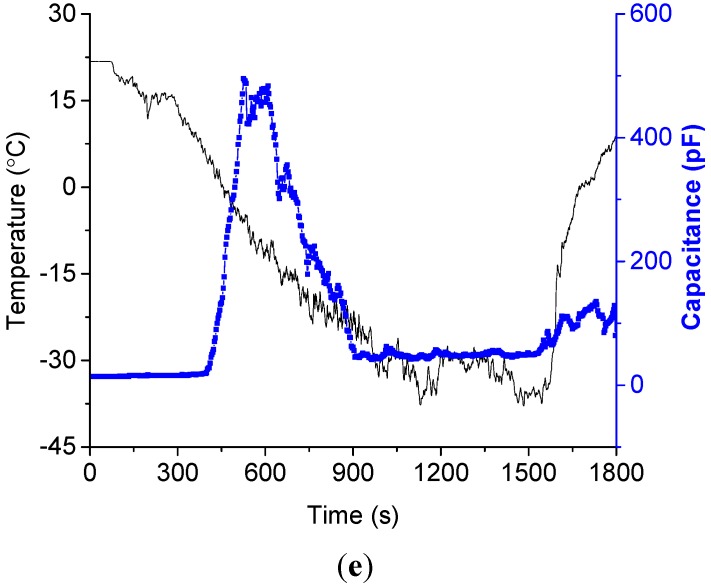
Real time measurement of ice growth for the different relative humidity: (**a**) at a relative humidity of 30%; (**b**) at a relative humidity of 35%; (**c**) at a relative humidity of 44%; (**d**) at a relative humidity of 52%; (**e**) at a relative humidity of 60%.

The measured capacitance was saturated under the condition of a continuous supply of water droplets. It is interesting to examine the variation of saturation temperature with respect to environmental humidity. Different relative humidities of 30%, 35%, 44%, 52%, and 60% were tested, as shown in Figure 10. As the humidity increased, the saturation temperature decreased. This could be explained by the number or possibility of the water droplets attaching to the sensor surface.

The second increase in the capacitance in the graph could again be explained by water film formation. The frost ice was melted by an air blower. The melted water formed a film on the ice sensor and, as a result, the capacitance increased.

The fabricated capacitive ice sensor measured the ice growth in real time. The measured capacitance provided meaningful information to indicate the existence of water in jet fuel and to alarm about possible ice growth. However, it could not simulate the ice growth from the fuel pipe surface because the frost ice did not grow only from the surface of the cold plate but also from the sensor surface. To improve this, the formation of the water film on the sensor surface must be prevented by using a hydrophobic treatment.

## 6. Conclusions

A measurement of the growth of ice thickness in real time was successfully performed using a capacitive ice sensor. Especially, the measured capacitance could explain the situation of ice formation. Contrary to expectations, as the water film on the sensor surface froze, the capacitance decreased and became saturated at different levels depending on the concentration of the water supply.

To simulate ice formation in jet fuel, not only the test environment but also the ice sensor should be improved to allow the ice growth only from the surface of the jet fuel pipe. One possible suggestion is to treat the surface of the ice sensor with a hydrophobic coating to prevent the attachment of water.

## References

[1] (2009). Safety Recommendation A-09-017 and A-09-018.

[2] Grade J.D., Jerman H., Kenny T.W. (2003). Design of Large Deflection Electrostatic Actuators. J. Microelectromech. Syst..

[3] Yazdi N., Kulah H., Najafi K. Precision Readout Circuits for Capacitive Microaccelerometers. Proceedings of IEEE Sensors 2004.

[4] Brank J., Yao J., Eberly M., Malczewski A., Varian K., Goldsmith C. (2001). RF MEMS-Based Tunable Filters. Int. J. RF Microw. Comput.-Aided Eng..

[5] Raymond A.S., John W.J. (2008). Physics for Scientists and Engineers with Modern Physics.

[6] Fujita S., Matsuoka T. A Summary of the Complex Dielectric Permittivity of Ice in the Megahertz Range and its Applications for Radar Sounding of Polar Ice Sheets. Proceedings of International Symposium on Physics of Ice Core Records.

[7] Technical Notes: Thermocouple Accuracy. http://www.microlink.co.uk/tctable.html.

[8] Rizzoni G. (2005). Principles and Applications of Electrical Engineering.

[9] Mattei E., Lauro S.E., Vannaroni G., Cosciotti B., Bella F., Pettinelli E. (2014). Dielectric Measurements and Radar Attenuation Estimation of Ice/Basalt Sand Mixtures as Martian Polar Caps Analogues. Icarus.

[10] Troiano A., Pasero E., Mesin L. (2011). New System for Detecting Road Ice Formation. IEEE Trans. Instrum. Meas..

